# Supplementary material for: Biophysically detailed mathematical models of multiscale cardiac active mechanics
Source: PLoS Comput Biol. 2020 Oct 7;16(10):e1008294. doi: 10.1371/journal.pcbi.1008294 (PMC7571720; doi:10.1371/journal.pcbi.1008294)
Supplement: S1 Appendix — This appendix contains the mathematical details about the formal derivation of the models proposed in this paper. (PDF) [file pcbi.1008294.s001.pdf]

Biophysically detailed mathematical models of multiscale cardiac active mechanics

Francesco Regazzoni[1*], Luca Dedè[1], Alfio Quarteroni[1,2]

1 MOX - Dipartimento di Matematica, Politecnico di Milano, P.zza Leonardo da Vinci 32, 20133 Milano, Italy
2 Mathematics Institute, École Polytechnique Fédérale de Lausanne, Av. Piccard, CH-1015 Lausanne, Switzerland (*Professor Emeritus*)

* francesco.regazzoni@polimi.it

S1 Appendix. Models derivation

We report the detailed derivation of the models proposed in the paper. The derivation is based on the assumptions discussed in the section Models. These assumptions allow to neglect second-order interactions among the stochastic processes, so that the variables can be partially decoupled, thus leading to drastic reductions in the size of model. Such strategy is illustrated in the following proposition.

Proposition 1. *Let* $(\Omega, \mathcal{A}, \mathbb{P})$ *be a probability space. Let* $A, B, C \subset \Omega$ *and let* $\mathcal{D}$ *be a finite partition of* Ω*, such that:*

(H1) $A \perp\!\!\!\perp B \,|\, C, D \quad \forall\, D \in \mathcal{D}$;

(H2) $B \perp\!\!\!\perp D \,|\, C \quad \forall\, D \in \mathcal{D}$.

Then, we have:

$$\mathbb{P}\left[A|B,C\right] = \frac{\sum_{D\in\mathcal{D}} \mathbb{P}\left[A|C,D\right]\mathbb{P}\left[C,D\right]}{\mathbb{P}\left[C\right]} = \mathbb{P}\left[A|C\right].$$

Proof. We have:

$$\begin{aligned}\mathbb{P}\left[A|B,C\right] &= \frac{\mathbb{P}\left[A,B,C\right]}{\mathbb{P}\left[B,C\right]} = \frac{\sum_{D\in\mathcal{D}} \mathbb{P}\left[A,B,C,D\right]}{\mathbb{P}\left[B,C\right]} = \frac{\sum_{D\in\mathcal{D}} \mathbb{P}\left[A|B,C,D\right]\mathbb{P}\left[B,C,D\right]}{\mathbb{P}\left[B,C\right]} \\ &= \frac{\sum_{D\in\mathcal{D}} \mathbb{P}\left[A|B,C,D\right]\mathbb{P}\left[B|C,D\right]\mathbb{P}\left[C,D\right]}{\mathbb{P}\left[B,C\right]}.\end{aligned}$$

From (H1), it follows:

$$\mathbb{P}\left[A|B,C,D\right] = \mathbb{P}\left[A|C,D\right].$$

Moreover, in virtue of (H2), we have:

$$\mathbb{P}\left[B|C,D\right] = \mathbb{P}\left[B|C\right] = \mathbb{P}\left[B,C\right]/\mathbb{P}\left[C\right].$$

By substituting into the above equation, the thesis follows. □

In the following, we will use several times the result of Prop. 1, where (H1) is a modeling choice on the dynamics of the system and (H2) is a simplifying assumption. Specifically, A is the target event, whose probability is the aim of the computation. In many situations, we know the joint probability of C and B, whereas the probability of A can be obtained by the joint probability of C and a different event D. Proposition 1 allows to pass from B to D, by assuming that the knowledge of B does not provide any further information when C and D are known.

Remark: the equations references in this document are referred to the main paper.

Definition of transition rates

As a starting point for a rigorous derivation of the proposed models, we provide a precise definition of the transition rates that govern the dynamics of the stochastic processes T_i^t, C_i^t, A_i^t, M_j^t and Z_i^t. Specifically, we have, for $\delta \in \{\mathcal{B},\mathcal{U}\}$ and $\alpha, \beta, \eta \in \{\mathcal{P},\mathcal{N}\}$:

$$
\begin{aligned}
k_{C,i}^{\delta\overline{\delta}|\beta} &= \lim_{\Delta t \to 0} \frac{1}{\Delta t} \mathbb{P}\left[C_i^{t+\Delta t} = \overline{\delta} \,|\, (C_i, T_i)^t = (\delta, \beta)\right], \\
k_{T,i}^{\beta\overline{\beta}|\alpha\,\cdot\,\eta,\delta} &= \lim_{\Delta t \to 0} \frac{1}{\Delta t} \mathbb{P}\left[T_i^{t+\Delta t} = \overline{\beta} \,|\, (T_{i-1}, T_i, T_{i+1}, C_i)^t = (\alpha, \beta, \eta, \delta)\right], \\
f_\alpha^i(x, v(t)) &= \lim_{\Delta t \to 0} \frac{1}{\Delta t} \mathbb{P}\left[Z_i^{t+\Delta t} = x \,|\, Z_i^t = \emptyset, T_i^t = \alpha, \right. \\
&\qquad\qquad \left. \exists\, j \in \mathcal{I}_M : d_{ij}^t = x + v_{\text{hs}}\Delta t, M_j^t = 0\right], \\
g_\alpha^i(x, v(t)) &= \lim_{\Delta t \to 0} \frac{1}{\Delta t} \mathbb{P}\left[Z_i^{t+\Delta t} = \emptyset \,|\, Z_i^t = x, T_i^t = \alpha\right],
\end{aligned}
$$

where $v(t)$ (i.e. the normalized shortening velocity) is assumed to be given. In the definition of f_α^i, the events conditioning the probability ensure that, at time t, the i-th BS is not attached and that there exists a non-attached MH at distance $x + v_{\text{hs}}\Delta t$ (so that at time $t + \Delta t$ the distance is reduced to x).

Derivation of Eq. (5)

Le us consider the time increment Δt and let us compute the probability $\pi_i^{\alpha\beta\delta,\vartheta\eta\lambda}(t + \Delta t)$. In virtue of the Bayes formula [1], we have:

$$
\begin{aligned}
&\pi_i^{\alpha\beta\delta,\vartheta\eta\lambda}(t + \Delta t) \overset{\Delta t \to 0}{\sim} \\
&\quad \mathbb{P}\left[T_{i-1}^{t+\Delta t} = \alpha | (T_{i-1}, T_i, T_{i+1})^t = (\overline{\alpha}, \beta, \delta), (C_{i-1}, C_i, C_{i+1})^t = (\vartheta, \eta, \lambda)\right] \pi_i^{\overline{\alpha}\beta\delta,\vartheta\eta\lambda}(t) \\
&\quad + \mathbb{P}\left[T_i^{t+\Delta t} = \beta | (T_{i-1}, T_i, T_{i+1})^t = (\alpha, \overline{\beta}, \delta), (C_{i-1}, C_i, C_{i+1})^t = (\vartheta, \eta, \lambda)\right] \pi_i^{\alpha\overline{\beta}\delta,\vartheta\eta\lambda}(t) \\
&\quad + \mathbb{P}\left[T_{i+1}^{t+\Delta t} = \delta | (T_{i-1}, T_i, T_{i+1})^t = (\alpha, \beta, \overline{\delta}), (C_{i-1}, C_i, C_{i+1})^t = (\vartheta, \eta, \lambda)\right] \pi_i^{\alpha\beta\overline{\delta},\vartheta\eta\lambda}(t) \\
&\quad + \mathbb{P}\left[C_{i-1}^{t+\Delta t} = \vartheta | (T_{i-1}, T_i, T_{i+1})^t = (\alpha, \beta, \delta), (C_{i-1}, C_i, C_{i+1})^t = (\overline{\vartheta}, \eta, \lambda)\right] \pi_i^{\alpha\beta\delta,\overline{\vartheta}\eta\lambda}(t) \\
&\quad + \mathbb{P}\left[C_i^{t+\Delta t} = \eta | (T_{i-1}, T_i, T_{i+1})^t = (\alpha, \beta, \delta), (C_{i-1}, C_i, C_{i+1})^t = (\vartheta, \overline{\eta}, \lambda)\right] \pi_i^{\alpha\beta\delta,\vartheta\overline{\eta}\lambda}(t) \\
&\quad + \mathbb{P}\left[C_{i+1}^{t+\Delta t} = \lambda | (T_{i-1}, T_i, T_{i+1})^t = (\alpha, \beta, \delta), (C_{i-1}, C_i, C_{i+1})^t = (\vartheta, \eta, \overline{\lambda})\right] \pi_i^{\alpha\beta\delta,\vartheta\eta\overline{\lambda}}(t) \\
&\quad + \mathbb{P}\left[(T_{i-1}, T_i, T_{i+1})^{t+\Delta t} = (\alpha, \beta, \delta), (C_{i-1}, C_i, C_{i+1})^{t+\Delta t} = (\vartheta, \eta, \lambda) | \right. \\
&\qquad \left. (T_{i-1}, T_i, T_{i+1})^t = (\alpha, \beta, \delta), (C_{i-1}, C_i, C_{i+1})^t = (\vartheta, \eta, \lambda)\right] \pi_i^{\alpha\beta\delta,\vartheta\eta\lambda}(t),
\end{aligned}
$$

where, by definition, we have:

$$
\mathbb{P}\left[T_i^{t+\Delta t} = \beta | (T_{i-1}, T_i, T_{i+1})^t = (\alpha, \overline{\beta}, \delta), (C_{i-1}, C_i, C_{i+1})^t = (\vartheta, \eta, \lambda)\right] \overset{\Delta t \to 0}{\sim} k_{T,i}^{\overline{\beta}\beta|\alpha\,\cdot\,\delta,\eta} \Delta t,
$$

and

$$
\begin{aligned}
\mathbb{P}\left[C_{i-1}^{t+\Delta t} = \vartheta | (T_{i-1}, T_i, T_{i+1})^t = (\alpha, \beta, \delta), (C_{i-1}, C_i, C_{i+1})^t = (\overline{\vartheta}, \eta, \lambda)\right] &\overset{\Delta t \to 0}{\sim} k_{C,i-1}^{\overline{\vartheta}\vartheta|\alpha} \Delta t, \\
\mathbb{P}\left[C_i^{t+\Delta t} = \eta | (T_{i-1}, T_i, T_{i+1})^t = (\alpha, \beta, \delta), (C_{i-1}, C_i, C_{i+1})^t = (\vartheta, \overline{\eta}, \lambda)\right] &\overset{\Delta t \to 0}{\sim} k_{C,i}^{\overline{\eta}\eta|\beta} \Delta t, \\
\mathbb{P}\left[C_{i+1}^{t+\Delta t} = \lambda | (T_{i-1}, T_i, T_{i+1})^t = (\alpha, \beta, \delta), (C_{i-1}, C_i, C_{i+1})^t = (\vartheta, \eta, \overline{\lambda})\right] &\overset{\Delta t \to 0}{\sim} k_{C,i+1}^{\overline{\lambda}\lambda|\delta} \Delta t.
\end{aligned}
$$

By adopting assumption (H1) and applying Prop. 1 for $A = (T_{i-1}^{t+\Delta t} = \overline{\alpha})$, $B = (T_{i+1}^t = \delta, C_{i+1}^t = \lambda)$, $C = ((T_{i-1}, T_i)^t = (\alpha, \beta), (C_{i-1}, C_i)^t = (\vartheta, \eta))$ and $\mathcal{D} = \{(T_{i-2}^t = \xi, C_{i-2}^t = \zeta)\}_{\xi,\zeta}$, we have:

$$\begin{aligned}
&\mathbb{P}\left[T_{i-1}^{t+\Delta t} = \alpha | (T_{i-1}, T_i, T_{i+1})^t = (\overline{\alpha}, \beta, \delta), (C_{i-1}, C_i, C_{i+1})^t = (\vartheta, \eta, \lambda)\right] \\
&\quad = \left(\sum_{\xi,\zeta} \pi_{i-1}^{\xi\overline{\alpha}\beta,\zeta\vartheta\eta}(t) \right)^{-1} \sum_{\xi,\zeta} \mathbb{P}\left[T_{i-1}^{t+\Delta t} = \alpha | (T_{i-2}, T_{i-1}, T_i)^t = (\xi, \overline{\alpha}, \beta), \right. \\
&\qquad\qquad \left. (C_{i-2}, C_{i-1}, C_i)^t = (\zeta, \vartheta, \eta)\right] \pi_{i-1}^{\xi\overline{\alpha}\beta,\zeta\vartheta\eta}(t) \\
&\quad = \frac{\sum_{\xi,\zeta} k_{T,i}^{\overline{\alpha}\alpha|\xi\,\cdot\,\beta,\vartheta} \pi_{i-1}^{\xi\overline{\alpha}\beta,\zeta\vartheta\eta}(t)}{\sum_{\xi,\zeta} \pi_{i-1}^{\xi\overline{\alpha}\beta,\zeta\vartheta\eta}(t)} \Delta t + o(\Delta t),
\end{aligned}$$

and similarly for the term related to $T_{i+1}^{t+\Delta t}$. In conclusion, by taking the limit $\Delta t \to 0$, we obtain Eq. (5).

Derivation of Eq. (15)

Before showing the derivation of Eq. (15), we precisely state the hypothesis of invariance by translation of the joint distribution of RUs. Specifically, we assume that, for any set of indices $\mathcal{I}_1 \subset \mathbb{Z}$ and $\mathcal{I}_2 \subset \mathbb{Z}$ and for any collection of states $\alpha_i \in \{\mathcal{N}, \mathcal{P}\}$ (for $i \in \mathcal{I}_1$) and $\beta_i \in \{\mathcal{U}, \mathcal{B}\}$ (for $i \in \mathcal{I}_2$), the joint distribution of the states of the corresponding RUs is not affected when the RUs are translated by a count of $k \in \mathbb{Z}$ units:

$$\mathbb{P}\left[\left(\bigcap_{i\in\mathcal{I}_1} T_i^t = \alpha_i\right) \cap \left(\bigcap_{i\in\mathcal{I}_2} C_i^t = \beta_i\right)\right] = \mathbb{P}\left[\left(\bigcap_{i\in\mathcal{I}_1} T_{i+k}^t = \alpha_i\right) \cap \left(\bigcap_{i\in\mathcal{I}_2} C_{i+k}^t = \beta_i\right)\right].$$

Similarly to what done before, we consider a finite time increment Δt and we write:

$$\begin{aligned}
\pi^{\alpha\beta\delta,\eta}(t+\Delta t) \overset{\Delta t\to 0}{\sim} &\mathbb{P}\left[T_{i-1}^{t+\Delta t} = \alpha | (T_{i-1}, T_i, T_{i+1})^t = (\overline{\alpha}, \beta, \delta), C_i^t = \eta\right] \pi^{\overline{\alpha}\beta\delta,\eta}(t) \\
&+\mathbb{P}\left[T_i^{t+\Delta t} = \beta | (T_{i-1}, T_i, T_{i+1})^t = (\alpha, \overline{\beta}, \delta), C_i^t = \eta\right] \pi^{\alpha\overline{\beta}\delta,\eta}(t) \\
&+\mathbb{P}\left[T_{i+1}^{t+\Delta t} = \delta | (T_{i-1}, T_i, T_{i+1})^t = (\alpha, \beta, \overline{\delta}), C_i^t = \eta\right] \pi^{\alpha\beta\overline{\delta},\eta}(t) \\
&+\mathbb{P}\left[C_i^{t+\Delta t} = \eta | (T_{i-1}, T_i, T_{i+1})^t = (\alpha, \beta, \delta), C_i^t = \overline{\eta}\right] \pi^{\alpha\beta\delta,\overline{\eta}}(t) \\
&+\mathbb{P}\left[(T_{i-1}, T_i, T_{i+1})^{t+\Delta t} = (\alpha, \beta, \delta), C_i^{t+\Delta t} = \eta | \right. \\
&\qquad \left. (T_{i-1}, T_i, T_{i+1})^t = (\alpha, \beta, \delta), C_i^t = \eta\right] \pi^{\alpha\beta\delta,\eta}(t),
\end{aligned}$$

where, by definition of the transition rates, it holds:

$$\mathbb{P}\left[T_i^{t+\Delta t} = \beta | (T_{i-1}, T_i, T_{i+1})^t = (\alpha, \overline{\beta}, \delta), C_i^t = \eta\right] \overset{\Delta t\to 0}{\sim} k_T^{\overline{\beta}\beta|\alpha\,\cdot\,\delta,\eta} \Delta t,$$

and

$$\mathbb{P}\left[C_i^{t+\Delta t} = \eta | (T_{i-1}, T_i, T_{i+1})^t = (\alpha, \beta, \delta), C_i^t = \overline{\eta}\right] \overset{\Delta t\to 0}{\sim} k_C^{\overline{\eta}\eta|\beta} \Delta t.$$

By adopting assumption (H4), Prop. 1 for $A = (T_{i-1}^{t+\Delta t} = \eta)$, $B = (T_{i+1}^t = \delta, C_i^t = \eta)$, $C = ((T_{i-1}, T_i)^t = (\alpha, \beta))$ and $\mathcal{D} = \{(T_{i-2}^t = \xi, C_{i-1}^t = \zeta)\}_{\xi,\zeta}$ leads to:

$$\begin{aligned}
&\mathbb{P}\left[T_{i-1}^{t+\Delta t} = \alpha | (T_{i-1}, T_i, T_{i+1})^t = (\overline{\alpha}, \beta, \delta), C_i^t = \eta\right] \\
&\quad = \frac{\sum_{\xi,\zeta} \mathbb{P}\left[T_{i-1}^{t+\Delta t} = \alpha | (T_{i-2}, T_{i-1}, T_i)^t = (\xi, \overline{\alpha}, \beta), C_{i-1}^t = \zeta\right] \pi_{i-1}^{\xi\overline{\alpha}\beta,\zeta}(t)}{\sum_{\xi,\zeta} \pi_{i-1}^{\xi\overline{\alpha}\beta,\zeta}(t)} \\
&\quad \overset{\Delta t\to 0}{\sim} \widetilde{k}_T^{\overline{\alpha}\alpha|\circ\,\cdot\,\beta,\circ} \Delta t.
\end{aligned}$$

In conclusion, by letting $\Delta t \to 0$, we get Eq. (15).

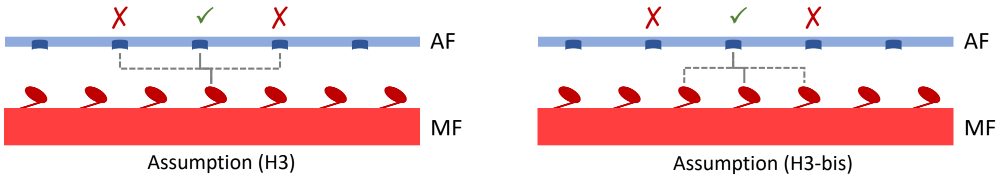

Fig A. Representation of assumptions (H3)-(H3-bis). According to assumption (H3) (respectively, assumption (H3-bis)) when a BS-MH pair is within the XB formation range, then the adjacent BSs (respectively, MHs) cannot be bound to the considered MH (respectively, BS).

Derivation of Eqs. (7) and (17)

We start with a remark on Ass. (H3).

Remark 1. Assumption (H3) states that, whenever a MH can bind to a given BS, it cannot be involved in a XB with another BS. Suppose that the support of f is contained in the interval $[x_1, x_1 + h]$. Then, this is equivalent to say that, if $d_{ij} \in [x_1, x_1 + h]$, the XBs between the couples $(i-1, j)$ and $(i+1, j)$, which feature displacements $d_{ij} - D_A$ and $d_{ij} + D_A$ respectively, cannot exist. This condition is automatically fulfilled if XBs are present only for displacements in the interval $(-D_A + x_1 + h, D_A + x_1)$, which has width $2D_A - h$. The interval consists in the support of f, with width h, surrounded by two bands of width $D_A - h$. Consider now the following condition:

$$f^i_{\mathcal{P}}(d_{ij}(t), v(t)) \neq 0 \implies M_k \neq i \quad \forall\, k \neq j. \tag{H3-bis}$$

Assumption (H3-bis) states that, whenever a BS lies within the attachment range of a given MH, it cannot be involved in a XB with another MH. By similar considerations as above, it turns out that this hypothesis is satisfied if XBs are present only in the range $(-D_M + x_1 + h, D_M + x_1)$. Since $D_M > D_A$, assumption (H3) is stronger than (H3-bis). Assumptions (H3)-(H3-bis) allow to decouple the dynamics of the different units. Their validity is justified when the shortening velocity is relatively small, whereas, for large velocities, the XB displacements may be convected outside the region $(-D_A + x_1 + h, D_A + x_1)$. Figure S1-1 provides a visual representation of assumptions (H3)-(H3-bis).

We recall that we have defined $d_{ij}(t)$ as the distance between the i-th actin BS and the j-th MH at time t. Since the myofilaments mutually slide with velocity $v_{\text{hs}}(t) = -\frac{d}{dt} SL(t)/2$, we have, for some constant d_0:

$$d_{ij}(t) = D_A i - D_M j + \frac{SL(t)}{2} - d_0,$$

D_A and D_M being the distance between two consecutive BSs and MHs, respectively. In order to account for the imperfections in the sarcomere lattice, we consider the value of d_0 as a random variable rather than a constant. Hence, we assume that, given a BS in front of the MF, the probability that the closest MH is located at distance x is uniform for $x \in [0, D_M)$. We denote by $\rho_M := \mathbb{f}\left[\exists\, j \in \mathbb{Z} \colon d^t_{ij} = x\right] = {D_M}^{-1}$ the MH linear density, that is:

$$\mathbb{P}\left[\exists\, j \in \mathbb{Z} \colon d^t_{ij} = x \in (a, b)\right] = \int_a^b \mathbb{f}\left[\exists\, j \in \mathbb{Z} \colon d^t_{ij} = x\right] dx = \rho_M |b - a|.$$

Let us consider now the variable $n_{i,\mathcal{P}}(x, t)$ (similar calculations can be carried out for

$n_{i,\mathcal{N}}(x,t))$. We have:

$$
\begin{aligned}
&\mathbb{f}\left[(Z_i,T_i)^{t+\Delta t} = (x - v_{\mathrm{hs}}(t)\Delta t,\mathcal{P})\right] \overset{\Delta t\to 0}{\sim} \\
&\qquad \mathbb{f}\left[(Z_i,T_i)^{t+\Delta t} = (x - v_{\mathrm{hs}}(t)\Delta t,\mathcal{P}) | (Z_i,T_i)^t = (\emptyset,\mathcal{P})\right]\mathbb{P}\left[(Z_i,T_i)^t = (\emptyset,\mathcal{P})\right] \\
&\quad + \mathbb{f}\left[(Z_i,T_i)^{t+\Delta t} = (x - v_{\mathrm{hs}}(t)\Delta t,\mathcal{P}) | (Z_i,T_i)^t = (x,\mathcal{N})\right]\mathbb{f}\left[(Z_i,T_i)^t = (x,\mathcal{N})\right] \\
&\quad + \mathbb{f}\left[(Z_i,T_i)^{t+\Delta t} = (x - v_{\mathrm{hs}}(t)\Delta t,\mathcal{P}) | (Z_i,T_i)^t = (x,\mathcal{P})\right]\mathbb{f}\left[(Z_i,T_i)^t = (x,\mathcal{P})\right].
\end{aligned}
$$

Thanks to Prop. 1, by taking $A = ((Z_i,T_i)^{t+\Delta t} = (x - v_{\mathrm{hs}}(t)\Delta t,\mathcal{P}))$, $B = (Z_i^t = x)$, $C = (T_i^t = \mathcal{N})$ and $\mathcal{D} = \{(T_{i-1},T_{i+1},C_i)^t = (\alpha,\eta,\delta)\}_{\alpha,\eta,\delta}$, assumption (H2) leads to

$$
\mathbb{f}\left[(Z_i,T_i)^{t+\Delta t} = (x - v_{\mathrm{hs}}(t)\Delta t,\mathcal{P}) | (Z_i,T_i)^t = (x,\mathcal{N})\right] \overset{\Delta t\to 0}{\sim} \widetilde{k}_{T,i}^{\mathcal{N}\mathcal{P}}\,\Delta t.
$$

where we have defined:

$$
\begin{aligned}
\widetilde{k}_{T,i}^{\mathcal{N}\mathcal{P}} &:= \frac{\sum_{\alpha,\eta,\delta} k_{T,i}^{\mathcal{N}\mathcal{P}|\alpha\,\cdot\,\eta,\delta}\,\mathbb{P}\left[(T_{i-1},T_i,T_{i+1},C_i)^t = (\alpha,\mathcal{N},\eta,\delta)\right]}{\mathbb{P}\left[T_i^t = \mathcal{N}\right]}, \\
\widetilde{k}_{T,i}^{\mathcal{P}\mathcal{N}} &:= \frac{\sum_{\alpha,\eta,\delta} k_{T,i}^{\mathcal{P}\mathcal{N}|\alpha\,\cdot\,\eta,\delta}\,\mathbb{P}\left[(T_{i-1},T_i,T_{i+1},C_i)^t = (\alpha,\mathcal{P},\eta,\delta)\right]}{\mathbb{P}\left[T_i^t = \mathcal{P}\right]}.
\end{aligned}
$$

We notice that the transition rates $\widetilde{k}_{T,i}^{\mathcal{N}\mathcal{P}}$ and $\widetilde{k}_{T,i}^{\mathcal{P}\mathcal{N}}$ can be obtained from the variables $\pi_i^{\alpha\beta\delta,\vartheta\eta\lambda}$ as in Eq. (8). Moreover, we have:

$$
\begin{aligned}
&\mathbb{P}\left[(Z_i,T_i)^{t+\Delta t} = (x - v_{\mathrm{hs}}(t)\Delta t,\mathcal{P}) | (Z_i,T_i)^t = (x,\mathcal{P})\right] \\
&\overset{\Delta t\to 0}{\sim} 1 - \mathbb{P}\left[(Z_i,T_i)^{t+\Delta t} = (\emptyset,\mathcal{P}) | (Z_i,T_i)^t = (x,\mathcal{P})\right] \\
&\qquad - \mathbb{P}\left[(Z_i,T_i)^{t+\Delta t} = (x - v_{\mathrm{hs}}(t)\Delta t,\mathcal{N}) | (Z_i,T_i)^t = (x,\mathcal{P})\right] \\
&\overset{\Delta t\to 0}{\sim} 1 - \Delta t\left(g_{\mathcal{P}}^i(x,v(t)) - \widetilde{k}_{T,i}^{\mathcal{P}\mathcal{N}}\right),
\end{aligned}
$$

where we have applied once again assumption (H2). Concerning the XB formation term, we have:

$$
\begin{aligned}
(F) &:= \mathbb{f}\left[(Z_i,T_i)^{t+\Delta t} = (x - v_{\mathrm{hs}}(t)\Delta t,\mathcal{P}) | (Z_i,T_i)^t = (\emptyset,\mathcal{P})\right]\mathbb{P}\left[(Z_i,T_i)^t = (\emptyset,\mathcal{P})\right] \\
&= \mathbb{f}\left[(Z_i,T_i)^{t+\Delta t} = (x - v_{\mathrm{hs}}(t)\Delta t,\mathcal{P}), (Z_i,T_i)^t = (\emptyset,\mathcal{P})\right] \\
&= \mathbb{f}\left[(Z_i,T_i)^{t+\Delta t} = (x - v_{\mathrm{hs}}(t)\Delta t,\mathcal{P}), (Z_i,T_i)^t = (\emptyset,\mathcal{P}), \exists\, j \in \mathcal{I}_M \colon d_{ij}^t = x, M_j^t = 0\right] \\
&+ \mathbb{f}\left[(Z_i,T_i)^{t+\Delta t} = (x - v_{\mathrm{hs}}(t)\Delta t,\mathcal{P}), (Z_i,T_i)^t = (\emptyset,\mathcal{P}), \exists\, j \in \mathcal{I}_M \colon d_{ij}^t = x, M_j^t \neq 0\right] \\
&+ \mathbb{f}\left[(Z_i,T_i)^{t+\Delta t} = (x - v_{\mathrm{hs}}(t)\Delta t,\mathcal{P}), (Z_i,T_i)^t = (\emptyset,\mathcal{P}), \exists\, j \in \mathbb{Z}\setminus\mathcal{I}_M \colon d_{ij}^t = x\right].
\end{aligned}
$$

The last two terms are at least of second order in Δt for $\Delta t \to 0$, while the first term gives:

$$
\begin{aligned}
&\mathbb{f}\left[(Z_i,T_i)^{t+\Delta t} = (x - v_{\mathrm{hs}}(t)\Delta t,\mathcal{P}), (Z_i,T_i)^t = (\emptyset,\mathcal{P}), \exists\, j \in \mathcal{I}_M \colon d_{ij}^t = x, M_j^t = 0\right] \\
&= \mathbb{P}\left[(Z_i,T_i)^{t+\Delta t} = (x - v_{\mathrm{hs}}(t)\Delta t,\mathcal{P}) | (Z_i,T_i)^t = (\emptyset,\mathcal{P}), \exists\, j \in \mathcal{I}_M \colon d_{ij}^t = x, M_j^t = 0\right] \\
&\quad \mathbb{f}\left[(Z_i,T_i)^t = (\emptyset,\mathcal{P}), \exists\, j \in \mathbb{Z} \colon d_{ij}^t = x, M_j^t = 0\right] \\
&\overset{\Delta t\to 0}{\sim} f_{\mathcal{P}}^i(x,v_{\mathrm{hs}}(t))\,\mathbb{f}\left[(Z_i,T_i)^t = (\emptyset,\mathcal{P}), \exists\, j \in \mathbb{Z} \colon d_{ij}^t = x, M_j^t = 0\right]\Delta t;
\end{aligned}
$$

the remaining two terms are null. Thus:

$$
(F) \sim f_{\mathcal{P}}^i(x,v(t))\,\Delta t\,\mathbb{f}\left[(Z_i,T_i)^t = (\emptyset,\mathcal{P}), \exists\, j \in \mathcal{I}_M \colon d_{ij}^t = x, M_j^t = 0\right].
$$

By assumption (H3), for any i and x such that $f^i_{\mathcal{P}}(x, v(t)) \neq 0$, the event $(M^t_j = 0)$ for j s.t. $d^t_{ij} = x$ implies the event $(Z^t_i = \emptyset)$, thus:

$$
\begin{aligned}
&\mathbb{f}\left[(Z_i, T_i)^t = (\emptyset, \mathcal{P}), \exists\, j \in \mathcal{I}_M \colon d^t_{ij} = x, M^t_j = 0\right] \\
&\quad = \mathbb{f}\left[(Z_i, T_i)^t = (\emptyset, \mathcal{P}), \exists\, j \in \mathcal{I}_M \colon d^t_{ij} = x\right] \\
&\quad = (\mathbb{f}\left[T^t_i = \mathcal{P}, \exists\, j \in \mathcal{I}_M \colon d^t_{ij} = x\right] \\
&\qquad - \sum_k \mathbb{f}\left[(Z_i, T_i)^t = (x + kD_M, \mathcal{P}), \exists\, j \in \mathcal{I}_M \colon d^t_{ij} = x\right]),
\end{aligned}
$$

since a BS can be only attached with displacements that are multiple of D_M. Moreover, we recall that the RU dynamics is independent of the interaction with XBs and thus of d_0 (see section Models) and that for i and x such that $f^i_{\mathcal{P}}(x, v(t)) \neq 0$ the events $(\exists\, j \in \mathcal{I}_M \colon d^t_{ij} = x)$ and $(\exists\, j \in \mathbb{Z} \colon d^t_{ij} = x)$ coincide. Therefore, we have (on the support of $f^i_{\mathcal{P}}$):

$$
\mathbb{f}\left[T^t_i = \mathcal{P}, \exists\, j \in \mathcal{I}_M\, d^t_{ij} = x\right] = \mathbb{P}\left[T^t_i = \mathcal{P}\right] \mathbb{f}\left[\exists\, j \in \mathbb{Z} \colon d^t_{ij} = x\right].
$$

In addition, since $(Z_i = x + kD_M)$ implies $(\exists\, j \in \mathbb{Z} \colon d^t_{ij} = x)$, on the support of $f^i_{\mathcal{P}}$ it holds true:

$$
\mathbb{f}\left[(Z_i, T_i)^t = (x + kD_M, \mathcal{P}), \exists\, j \in \mathcal{I}_M \colon d^t_{ij} = x\right] = \mathbb{f}\left[(Z_i, T_i)^t = (x + kD_M, \mathcal{P})\right].
$$

Since assumption (H3) implies (H3-bis), the unique nonzero term of the sum is $k = 0$ and thus:

$$
(F) \sim= f^i_{\mathcal{P}}(x, v(t)) \Delta t (\mathbb{P}\left[T^t_i = \mathcal{P}\right] \mathbb{f}\left[\exists\, j \in \mathbb{Z} \colon d^t_{ij} = x\right] - \mathbb{f}\left[(Z_i, T_i)^t = (x, \mathcal{P})\right]).
$$

Finally, we divide everything by Δt we let $\Delta t \to 0$ and we observe that:

$$
\begin{aligned}
&\frac{n_{i,\mathcal{P}}(x - v_{\text{hs}}(t)\Delta t, t + \Delta t) - n_{i,\mathcal{P}}(x, t)}{\Delta t} \\
&\quad = \frac{n_{i,\mathcal{P}}(x - v_{\text{hs}}(t)\Delta t, t + \Delta t) - n_{i,\mathcal{P}}(x - v_{\text{hs}}(t)\Delta t, t)}{\Delta t} \\
&\quad + \frac{n_{i,\mathcal{P}}(x - v_{\text{hs}}(t)\Delta t, t) - n_{i,\mathcal{P}}(x, t)}{\Delta t\, v_{\text{hs}}(t)} v_{\text{hs}}(t) \\
&\quad \to \frac{\partial n_{i,\mathcal{P}}}{\partial t}(x, t) - v_{\text{hs}}(t) \frac{\partial n_{i,\mathcal{P}}}{\partial x}(x, t).
\end{aligned}
$$

We get in such a way Eq. (7). Moreover, the expected value of the force exerted by the whole half filament is given by:

$$
\begin{aligned}
F_{\text{hf}}(t) &= \sum_i \int_{-\infty}^{+\infty} F_{\text{XB}}(x) \mathbb{f}\left[Z^t_i = x\right] dx \\
&= \sum_i \int_{-\infty}^{+\infty} F_{\text{XB}}(x) \left(\mathbb{f}\left[Z^t_i = x, T^t_i = \mathcal{P}\right] + \mathbb{f}\left[Z^t_i = x, T^t_i = \mathcal{N}\right]\right) dx \\
&= \sum_i \int_{-\infty}^{+\infty} F_{\text{XB}}(x) \left(n_{i,\mathcal{P}}(x, t) + n_{i,\mathcal{N}}(x, t)\right) dx.
\end{aligned}
$$

On the other hand, Eq. (17) can be derived similarly to Eq. (7), by dropping the dependence on the RU index i.

Derivation of Eqs. (11) and (21)

By following [2], we multiply Eq. (7) by $(\frac{x}{SL_0/2})^p$, for $p = 0, 1$, and we integrate with respect to x over the real line. Thanks to the fact that, for $x \to \pm\infty$, the distributions $n_{i,\alpha}$ are definitively equal to zero, for $\alpha \in \{\mathcal{N}, \mathcal{P}\}$ and for $i \in \mathcal{I}_A$, the convective terms give raise to the following terms. For $p = 0$, we have:

$$\int_{-\infty}^{+\infty} v_{\text{hs}} \frac{\partial n_{i,\alpha}}{\partial x} dx = [n_{i,\alpha}]_{-\infty}^{+\infty} = 0.$$

On the other hand, for $p = 1$, we have:

$$\int_{-\infty}^{+\infty} \frac{x}{SL_0/2} v_{\text{hs}} \frac{\partial n_{i,\alpha}}{\partial x} dx = -\int_{-\infty}^{+\infty} \frac{v_{\text{hs}}}{SL_0/2} n_{i,\mathcal{P}} dx + v_{\text{hs}} \left[\frac{x}{SL_0/2} n_{i,\alpha} \right]_{-\infty}^{+\infty} = -v\, \mu_{i,\alpha}^0(t).$$

Hence, simple calculations lead to Eqs. (11) and (21).

References

1. Norris JR. Markov Chains. 2. Cambridge University Press; 1998.
2. Zahalak GI. A distribution-moment approximation for kinetic theories of muscular contraction. Mathematical Biosciences. 1981;55(1-2):89–114.
