# Supplementary material for: Biophysically detailed mathematical models of multiscale cardiac active mechanics
Source: PLoS Comput Biol. 2020 Oct 7;16(10):e1008294. doi: 10.1371/journal.pcbi.1008294 (PMC7571720; doi:10.1371/journal.pcbi.1008294)
Supplement: S2 Appendix — This appendix provides further details about the calibration pipeline of the proposed models. (PDF) [file pcbi.1008294.s002.pdf]

Biophysically detailed mathematical models of multiscale cardiac active mechanics

Francesco Regazzoni[1*], Luca Dedè[1], Alfio Quarteroni[1,2]

1 MOX - Dipartimento di Matematica, Politecnico di Milano, P.zza Leonardo da Vinci 32, 20133 Milano, Italy
2 Mathematics Institute, École Polytechnique Fédérale de Lausanne, Av. Piccard, CH-1015 Lausanne, Switzerland (*Professor Emeritus*)

* francesco.regazzoni@polimi.it

S2 Appendix. Parameters calibration

To calibrate the parameters of the proposed models, we will restrict ourselves to experimental measurements from the literature coming from intact cardiac cells since the skinning procedure alters in a significant (and only partially understood) manner the activation and force generation dynamics [1–3]. Moreover, thanks to the technique of flura-2 fluorescence, it is nowadays possible to measure the intracellular calcium concentration without depriving the cell of its membrane, and it is also possible to inhibit the sarcoplasmic reticulum calcium uptake by cyclopiazonic acid, so that the calcium level can be controlled without the need of skinning the cells [3, 4].

Calibration of the XBs rates

In [5] we have shown that the parameters of the distribution-moments equations describing the XB dynamics can be calibrated starting from five quantities, that are described in the following. Under isometric conditions, we consider the isometric tension $T_\mathrm{a}^\mathrm{iso} := a_\mathrm{XB}\mu^1$ and the fraction of attached XBs $\mu^0_\mathrm{iso} := \mu^0$, where μ^0 and μ^1 are the steady-state solution for $v = 0$. The force-velocity relationship is characterized by the maximum shortening velocity v^max, and by v^0, its intersection with the axis $T_\mathrm{a} = 0$ of the tangent of the curve in isometric conditions, defined as:

$$v^0 := -\left(\left. \frac{\partial \overline{T}_\mathrm{a}(v)/T_\mathrm{a}^\mathrm{iso}}{\partial v} \right|_{v=0} \right)^{-1},$$

where $\overline{T}_\mathrm{a}(v)$ denotes the steady-state tension for velocity v. Finally, as discussed in [5], the response to fast inputs is characterized, in the small-velocity regime, by the normalized slope of the tension-elongation curve after a fast step. Such quantity is defined as follows: by applying a step in length ΔL to an isometrically contracted muscle in a small time interval Δt, we define by $T_\mathrm{a}(\Delta t)$ the tension recorded after the step is applied, and we define the normalized stiffness as:

$$\tilde{k}_2 := - \left. \frac{\partial T_\mathrm{a}(\Delta t)/T_\mathrm{a}^\mathrm{iso}}{\partial \Delta L} \right|_{\Delta L = 0}.$$

As discussed in [5], when the small-velocity regimes is considered, the quantity $\tilde{k}_2$ corresponds to the slope of the T_2-L_2 relationship.

Parameter	Value	Units	Reference
$T_{\mathrm{a}}^{\mathrm{iso}}$	120	kPa	[4]
μ_{iso}^{0}	0.22	-	[8]
$v^{\max}$	8	s^{-1}	[9]
v^{0}	2	s^{-1}	[9]
$\tilde{k}_2$	66	-	[9]

Table A. List of the experimental data used for model calibration.

In conclusion, as shown in [5], by acting on the five parameters $\mu_{f_{\mathcal{P}}}^{0}$, $\mu_{f_{\mathcal{P}}}^{1}$, r_0, α and a_{XB} we can fit experimentally measured values concerning the isometric solution ($T_{\mathrm{a}}^{\mathrm{iso}}$ and μ_{iso}^{0}), the force-velocity relationship ($v^{\max}$ and v^{0}) and the fast transients response ($\tilde{k}_2$). All the above mentioned experimental setups are such that the thin filament activation machinery can be considered in steady-state. Indeed, $[\mathrm{Ca}^{2+}]_{\mathrm{i}}$ is constant in all the cases and, concerning SL: it is also constant under isometric conditions; constant shortening experiments are typically performed in the plateau region of the force-length relationship, and thus the effect of changes in SL is irrelevant; fast transient experiments are carried out at a time-scale such that the activation variables can be considered constant, since they are characterized by a much slower dynamics [6, 7]. Therefore, in these cases, the values of P_i, $\widetilde{k}_{T,i}^{\mathcal{PN}}$ and $\widetilde{k}_{T,i}^{\mathcal{NP}}$ can be considered as fixed in Eq. (11) (and similarly in Eq. (21)).

We notice that, while for the models considered in [5] the relationship between the five parameters and the five experimentally measured values can be analytically inverted, in this case we find the values of the parameters with a numerical strategy. Specifically, to find the steady-state solution we solve Eq. (11) by setting to zero the time derivative terms; we consider the exact solution after the fast transient (the linear ODE system (11) can be solved analytically); we approximate the derivative appearing in the definition of v^0 and $\tilde{k}_2$ by finite differences. Finally, we solve, for the five parameters, the nonlinear system of equations linking the five measured values with the parameters themselves. With this aim we employ the Newton-Raphson method, by approximating the Jacobian matrix by means of finite differences.

The experimental data used to calibrate the model are reported in Table S2-1, together with a reference to the source in literature. As we mentioned before, we employ data coming from room-temperature intact cardiac rat cell, apart from μ_{iso}^{0} (acquired from skeletal frog muscle), for which we did not find measurements from cardiac muscles. However, as shown in [5], this variable only affects the value of the microscopic variables (i.e. $\mu_{i,\alpha}^{p}$), but not that of the predicted active tension T_{a}.

We notice that the constants $v^{\max}$, v^0 and $\tilde{k}_2$ are normalized with respect to $T_{\mathrm{a}}^{\mathrm{iso}}$ and are thus valid for a wide range of activation levels (see Introduction). Conversely, the value of $T_{\mathrm{a}}^{\mathrm{iso}}$ is associated with a SL in the plateau region and to saturating calcium concentration. Therefore, when we calibrate the parameters we set $[\mathrm{Ca}^{2+}]_{\mathrm{i}} = 10\,\mu\mathrm{M}$ and $SL = 2.2\,\mu\mathrm{m}$.

Calibration of the RUs rates (steady-state)

The steady-state characterization of the muscle tissue activation is represented by the force-calcium and force-length relationships (see Introduction), whose main features are the behavior for SL in the plateau region (characterized by the tension for saturating calcium $T_{\mathrm{a}}^{\mathrm{iso}}$, the calcium sensitivity EC_{50} and the cooperativity coefficient n_H) and the effect of SL (on the saturating tension $T_{\mathrm{a}}^{\mathrm{iso}}$ and on the calcium sensitivity EC_{50}).

We recall that, thanks to our strategy, the tension for saturating calcium concentrations $T_{\mathrm{a}}^{\mathrm{iso}}$ in the plateau region of SL is automatically fitted. The effect of k_{d}

is that of shifting the force-calcium curves with respect to the $\log [\mathrm{Ca}^{2+}]_\mathrm{i}$ axes since it only appears in combination with $[\mathrm{Ca}^{2+}]_\mathrm{i}$ in the model equations. Therefore, the value of k_d can be easily calibrated to match the experimental data as it only affects EC_{50}. The effect of γ, on the other hand, is that of tuning the amount of cooperativity (indeed, it acts on n_H). The role of the remaining parameters (Q and μ) is more involved and cannot be easily decoupled, as they affect the cooperativity, calcium sensitivity, and the asymmetry of the force-calcium relationship below and above EC_{50} [10]. Moreover, in the SE-ODE case, they also act on the SL-driven regulation on calcium sensitivity.

In the following we set $\mu = 10$, coherently with the fact that the experimentally measured dissociation rate of Tn from calcium varies of one order of magnitude in different combinations [11]. For the SE-ODE model, we set γ, Q and k_d, to fit the steady-state force-calcium measurements of [4] (referred to the two different values of SL of 1.85 and 2.15 µm) from intact rat cardiac cells at room temperature.

Calibration of the RUs rates (kinetics)

We consider the isometric twitches of intact rat cardiac muscle fibers reported in [12] for different values of SL, and with $[\mathrm{Ca}^{2+}]_\mathrm{o} = 1.0\,\mathrm{mM}$. Since the corresponding calcium transients are not reported, we consider the calcium transient reported in [13] for the same muscle preparation. As the reported trace is much affected by noise, we fit it with the following idealized transient [14]:

$$[\mathrm{Ca}^{2+}]_\mathrm{i}(t) = \begin{cases} c_0 & t < t_0^c, \\ c_0 + \frac{c_{\max} - c_0}{\beta} \left[e^{-\frac{t - t_0^c}{\tau_1^c}} - e^{-\frac{t - t_0^c}{\tau_2^c}} \right] & t \geq t_0^c\,, \end{cases}$$

where

$$\beta = \left(\frac{\tau_1^c}{\tau_2^c}\right)^{-\left(\frac{\tau_1^c}{\tau_2^c} - 1\right)^{-1}} - \left(\frac{\tau_1^c}{\tau_2^c}\right)^{-\left(1 - \frac{\tau_2^c}{\tau_1^c}\right)^{-1}},$$

with the constants values $c_{\max} = 1.35\,\mu\mathrm{M}$, $t_0^c = 0.05\,\mathrm{s}$, $\tau_1^c = 0.02\,\mathrm{s}$, $\tau_2^c = 0.19\,\mathrm{s}$.

Then, we sample the candidate parameters space $(k_\mathrm{basic}, k_\mathrm{off}) \in [0, 80\,\mathrm{s}^{-1}] \times [0, 300\,\mathrm{s}^{-1}]$ by a MC strategy, for each parameters value we compute the tension transients corresponding to $SL = 1.90$, 2.05 and 2.20 µm and we compare them with the experimental measurements from [12]. We consider the following discrepancy metrics, where $T_\mathrm{a}^{i,\mathrm{mod}}(t)$ denotes the tension predicted by the model for the i-th value of SL and $T_\mathrm{a}^{i,\mathrm{exp}}(t)$ denotes the experimentally measured one:

$$E_{L^2} := \sqrt{\sum_{i=1}^{3} \int_0^T \left| T_\mathrm{a}^{i,\mathrm{mod}}(t) - T_\mathrm{a}^{i,\mathrm{exp}}(t) \right|^2 dt},$$

$$E_\mathrm{peak} := \sqrt{\sum_{i=1}^{3} \left| \sup_{t \in [0,T]} T_\mathrm{a}^{i,\mathrm{mod}}(t) - \sup_{t \in [0,T]} T_\mathrm{a}^{i,\mathrm{exp}}(t) \right|^2}.$$

The first metric coincides with the L^2 error over time, while the second one evaluates the error of the predicted force peak. We notice indeed that the parameters k_basic and k_off also determine the force peak attained during a sarcomere twitch: the most rapid the tissue activation is, the more the force-calcium curve stays close to the steady-state curve and thus it reaches higher force values before the relaxation begins. As criterion to select the best parameters values, we consider as overall metric a weighted combination between the two, given by $E_\mathrm{tot} = (E_{L^2}^2 + w_\mathrm{peak}^2 E_\mathrm{peak}^2)^{1/2}$, where we set $w_\mathrm{peak} = 5$.

The obtained values of the discrepancy metric E_tot in the parameters space for both the SE-ODE and the MF-ODE models are reported in the below picture.

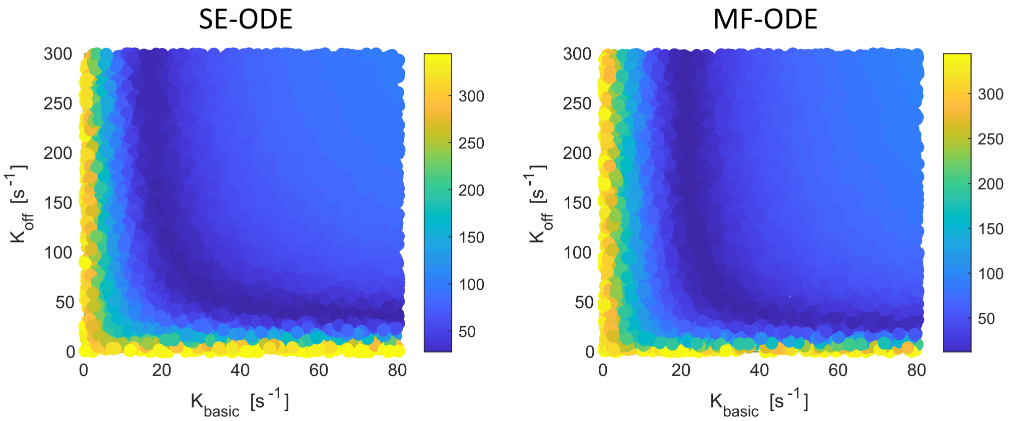

Fig A. Discrepancy metric E_{tot} in the parameters space for intact, room-temperature rat cells, obtained with the SE-ODE model (left) and with the MF-ODE model (right).

We notice that the level curves do not clearly identify an optimal pair $(k_{\text{basic}}, k_{\text{off}})$, rather these exhibit a wide region in the parameters space producing very similar results. Given the uncertainty in the measurements of both force and, mostly, calcium, it makes no sense to select the best parameters by blindly selecting the pair that realizes the smaller discrepancy from experimental results. Therefore, we supplement the results of Fig. S2-1 with direct measurements of calcium binding rates to Tn, showing that $k_{\text{on}} = k_{\text{off}}/k_{\text{d}}$ lies between 50 and $200\,\mu\text{M}^{-1}\,\text{s}^{-1}$ [11]. On this basis, we restrict the region of candidate values and we select the parameters reported in Tab. 3.

The predicted isometric twitches obtained with the selected values of the parameters are compared with the experimental data in Fig. 15. We notice here that the MF-ODE model accomplishes a better fit of experimental data than the SE-ODE model. This is an effect of the phenomenological tuning of k_{d} of Eq. (29), that allows for a significant increase of calcium sensitivity and, consequently, of twitch duration, for higher values of SL. Nonetheless, also the SE-ODE model predicts, even if to a lower extent, the experimentally observed prolongation of twitches at higher SL, without any phenomenological tuning of the calcium sensitivity.

We notice that there is room for improvement in the calibration of the kinetic parameters k_{basic} and k_{off}, which could be better constrained in presence of more abundant and more reliable experimental data and when a deeper understanding on the determinants of the kinetics of activation and relaxation will be available. Nevertheless, the calibration of k_{basic} and k_{off} for the rat model does not affect the quality of the human model, since those two parameters are the only ones to be completely re-calibrated for the human model.

Human model at body temperature

In order to adapt the parameters calibrated from rat data at room temperature to a body-temperature human model, we first focus on the steady state, to reflect a higher calcium sensitivity. For this purpose, we employ the data reported in [15], which, however, refer to skinned cells. In order to estimate the effect of skinning on k_{d}, we compare the calcium sensitivity measured for room-temperature rat cardiac cells in skinned [16] and intact preparations [4] at $SL = 2.15\,\mu\text{m}$ and we assume that the same relationship holds for skinned versus intact, body-temperature human cells. Finally, we rescale the values of k_{d} to reflect the estimated change in calcium sensitivity between intact, body-temperature human cells and intact, room-temperature rat cells, obtaining

Species	Temperature	Preparation	SL(µm)	EC_{50}(µM)	Reference
Rat	Room	Skinned	2.15	3.51	[16]
Rat	Room	Intact	2.15	0.68	[4]
Human	Body	Skinned	2.00	1.72	[15]
Human	Body	Skinned	2.20	1.56	[15]

Table B. List of the experimental values used to calibrate the calcium sensitivity for the human models at body temperature.

the values reported in Tab. 3. The experimental data used in such procedure are listed in Tab. S2-2.

Since the RUs kinetics may depend on both the species and the temperature, we re-calibrate the parameters k_{off} and k_{basic} on the base of the kinetic metrics reported in [17] (the data are referred to body-temperature human cells). These metrics include the peak tension $T_{\mathrm{a}}^{\mathrm{peak}}$, the time-to-peak TTP (defined as the time separating the beginning of force raise and the tension peak) and the relaxation times RT_{50} and RT_{95} (defined as the time between the tension peak and 50% and 95% of relaxation, respectively). Since, as to the best of our knowledge, detailed calcium transients measurements for intact human cells at body temperature are not currently available, we employ the synthetic calcium transient predicted by the ToR-ORd model [18]. The metrics reported in [17] are referred to different values of SL, expressed as fraction of the *optimal sarcomere length* (i.e. the length for which an increase of developed force is compensated by an increase in resting tension), corresponding, according to the authors, to nearly $SL = 2.2\,\mu\mathrm{m}$. For the calibration, we employ the value associated with 95% of the optimal length (i.e. 2.09 µm).

Finally, for the calibration of the parameters ruling the XBs cycling, we use the same values of Tab. S2-1. Therefore, since the calibration depends on the parameters previously set for the RUs activation, the resulting values of the parameters are slightly different. We provide in Tab. 3 the full list of parameters for both species (room-temperature rat and body-temperature human) and for both models (SE-ODE and MF-ODE).

References

1. Kentish JC, ter Keurs HEDJ, Ricciardi L, Bucx JJJ, Noble MIM. Comparison between the sarcomere length-force relations of intact and skinned trabeculae from rat right ventricle. Influence of calcium concentrations on these relations. Circulation Research. 1986;58(6):755–768.

2. Backx PH, Gao W, Azan-Backx MD, Marbán E. The relationship between contractile force and intracellular [Ca2+] in intact rat cardiac trabeculae. The Journal of General Physiology. 1995;105(1):1–19.

3. Ter Keurs HEDJ, Shinozaki T, Zhang YM, Zhang ML, Wakayama Y, Sugai Y, et al. Sarcomere mechanics in uniform and non-uniform cardiac muscle: a link between pump function and arrhythmias. Progress in biophysics and molecular biology. 2008;97(2-3):312–331.

4. Ter Keurs HEDJ, Hollander EH, ter Keurs MHC. The effect of sarcomere length on the force–cytosolic [Ca2+] relationship in intact rat cardiac trabeculae. Skeletal muscle mechanics: from mechanics to function Wiley, New York. 2000; p. 53–70.

5. Regazzoni F, Dedè L, Quarteroni A. Active force generation in cardiac muscle cells: mathematical modeling and numerical simulation of the actin-myosin interaction. Vietnam Journal of Mathematics. 2020;.

6. Katz AM. Physiology of the heart. Lippincott Williams & Wilkins; 2010.

7. Bers D. Excitation-contraction coupling and cardiac contractile force. vol. 237. Springer Science & Business Media; 2001.

8. Brunello E, Caremani M, Melli L, Linari M, Fernandez-Martinez M, Narayanan T, et al. The contributions of filaments and cross-bridges to sarcomere compliance in skeletal muscle. The Journal of Physiology. 2014;592(17):3881–3899.

9. Caremani M, Pinzauti F, Reconditi M, Piazzesi G, Stienen GJ, Lombardi V, et al. Size and speed of the working stroke of cardiac myosin in situ. Proceedings of the National Academy of Sciences. 2016;113(13):3675–3680.

10. Rice JJ, Stolovitzky G, Tu Y, de Tombe PP. Ising model of cardiac thin filament activation with nearest-neighbor cooperative interactions. Biophysical Journal. 2003;84(2):897–909.

11. Niederer SA, Hunter PJ, Smith NP. A quantitative analysis of cardiac myocyte relaxation: a simulation study. Biophysical Journal. 2006;90(5):1697–1722.

12. Janssen PM, Hunter WC. Force, not sarcomere length, correlates with prolongation of isosarcometric contraction. American Journal of Physiology - Heart and Circulatory Physiology. 1995;269(2):H676–H685.

13. Janssen PM, de Tombe PP. Uncontrolled sarcomere shortening increases intracellular Ca2+ transient in rat cardiac trabeculae. American Journal of Physiology - Heart and Circulatory Physiology. 1997;272(4):H1892–H1897.

14. Washio T, Okada J, Sugiura S, Hisada T. Approximation for cooperative interactions of a spatially-detailed cardiac sarcomere model. Cellular and Molecular Bioengineering. 2012;5(1):113–126.

15. Land S, Park-Holohan S, Smith NP, dos Remedios CG, Kentish JC, Niederer SA. A model of cardiac contraction based on novel measurements of tension development in human cardiomyocytes. Journal of Molecular and Cellular Cardiology. 2017;106:68–83.

16. Dobesh DP, Konhilas JP, de Tombe PP. Cooperative activation in cardiac muscle: impact of sarcomere length. American Journal of Physiology-Heart and Circulatory Physiology. 2002;51(3):H1055.

17. Chung JH, Martin BL, Canan BD, Elnakish MT, Milani-Nejad N, Saad NS, et al. Etiology-dependent impairment of relaxation kinetics in right ventricular end-stage failing human myocardium. Journal of molecular and cellular cardiology. 2018;121:81–93.

18. Tomek J, Bueno-Orovio A, Passini E, Zhou X, Minchole A, Britton O, et al. Development, calibration, and validation of a novel human ventricular myocyte model in health, disease, and drug block. Elife. 2019;8:e48890.
