# Supplementary material for: Biophysically detailed mathematical models of multiscale cardiac active mechanics
Source: PLoS Comput Biol. 2020 Oct 7;16(10):e1008294. doi: 10.1371/journal.pcbi.1008294 (PMC7571720; doi:10.1371/journal.pcbi.1008294)
Supplement: S3 Appendix — This appendix provides details about the numerical schemes employed to approximate the solution of the models proposed in this paper. (PDF) [file pcbi.1008294.s003.pdf]

Biophysically detailed mathematical models of multiscale cardiac active mechanics

Francesco Regazzoni[1*], Luca Dedè[1], Alfio Quarteroni[1,2]

1 MOX - Dipartimento di Matematica, Politecnico di Milano, P.zza Leonardo da Vinci 32, 20133 Milano, Italy
2 Mathematics Institute, École Polytechnique Fédérale de Lausanne, Av. Piccard, CH-1015 Lausanne, Switzerland (*Professor Emeritus*)

* francesco.regazzoni@polimi.it

S3 Appendix. Numerical schemes

We provide details about the numerical schemes employed to approximate the solution of the models proposed in this paper.

Remark: the equation references in this document are referred to the main paper.

RU dynamics

Since the equations describing the evolution of the RUs are independent of the variables describing the XB states, their solution can be approximated independently of that of the models describing the XB dynamics. Specifically, we adopt a Forward Euler scheme with a time step size of $2.5 \cdot 10^{-5}$ s. Further details on the properties of the numerical solution obtained by applying this scheme can be found in [1].

XB dynamics

Concerning the equations describing the XB dynamics, we notice that Eqs. (11) and (21) can be written in the following form:

$$\begin{cases} \dot{\mathbf{x}}(t) = \mathbf{A}(t)\mathbf{x}(t) + \mathbf{r}(t) & t \in (0, T], \\ \mathbf{x}(0) = \mathbf{x}_0, \end{cases} \tag{S2-1}$$

where $\mathbf{x}(t)$ is the vector of the variables describing the states of XBs, while $\mathbf{A}(t)$ and $\mathbf{r}(t)$ are respectively a time-dependent matrix and vector, determined by the input $v(t)$ and by the RUs states $\pi_i^{\alpha\beta\delta,\vartheta\eta\lambda}(t)$ (or, for mean-field models, $\pi^{\alpha\beta\delta,\eta}(t)$).

In order to approximate the solution of Eq. (S2-1), we consider a subdivision $0 = t_0 < t_1 < \cdots < t_M = T$ of the time interval $[0, T]$ with time step size Δt and we denote by $\mathbf{x}^{(k)} \approx \mathbf{x}(t_k)$ the approximated solution at time t_k. Due to the linearity of Eq. (S2-1), we consider the following exponential scheme [2]:

$$\begin{cases} \mathbf{x}^{(0)} = \mathbf{x}_0, & \\ \mathbf{x}_\infty^{(k)} = -\mathbf{A}^{-1}(t_k)\mathbf{r}(t_k) & \text{for } k \geq 1, \\ \mathbf{x}^{(k)} = \mathbf{x}_\infty^{(k)} + e^{\Delta t \mathbf{A}(t_k)}(\mathbf{x}^{(k-1)} - \mathbf{x}_\infty^{(k)}) & \text{for } k \geq 1. \end{cases} \tag{S2-2}$$

Due to the implicit nature of the scheme of Eq. (S2-2), that entails better stability properties than the explicit scheme used for the RUs equations [3], we solve it with a larger time step size than the one used for the RUs model ($\Delta t = 1 \cdot 10^{-3}$ s), by a first-order time splitting scheme.

References

1. Regazzoni F. Mathematical modeling and Machine Learning for the numerical simulation of cardiac electromechanics. PhD Thesis (Politecnico di Milano); 2020.

2. Hochbruck M, Ostermann A. Exponential integrators. Acta Numerica. 2010;19:209–286.

3. Quarteroni A, Sacco R, Saleri F. Numerical Mathematics. vol. 37. Springer Science & Business Media; 2010.
